# High survival of mouse oocytes using an optimized vitrification protocol

**DOI:** 10.1038/srep19465

**Published:** 2016-01-19

**Authors:** Cheng-Jie Zhou, Dong-Hui Wang, Xin-Xin Niu, Xiang-Wei Kong, Yan-Jiao Li, Jing Ren, Hong-Xia Zhou, Angeleem Lu, Yue-Fang Zhao, Cheng-Guang Liang

**Affiliations:** 1 The Key Laboratory of National Education, Ministry for Mammalian Reproductive Biology and Biotechnology, Inner Mongolia University, Hohhot, Inner Mongolia, People’s Republic of China

## Abstract

The method of vitrification has been widely used for cryopreservation. However, the effectiveness of this method for mammalian oocytes could be improved by optimizing each step of the process. In the present study, we tested the effects of varying several key parameters to determine the most effective protocol for mouse oocyte vitrification. We found that cryoprotectant containing ethylene glycol and dimethylsulfoxide plus 20% fetal calf serum produced the highest rates of oocyte survival, fertilization, and blastocyst formation. The duration and temperature of oocyte exposure to vitrification and thawing solutions influenced survival rate. The presence of cumulus cells surrounding oocytes and the incubation of thawed oocytes in Toyoda-Yokoyama-Hosoki medium also increased oocyte survival. Open pulled straw and nylon loop methods were more effective than the mini-drop method. Finally, the combination of these improved methods resulted in better spindle morphology when compared to the unimproved methods. These results demonstrate that the outcomes of mouse oocyte vitrification can be improved by a suitable combination of cryopreservation methods, which could be applied to future clinical research with human oocytes.

Gamete cryopreservation has become the leading technology for long-term germplasm preservation. For mammalian oocytes, the method of vitrification has been used for several species, including mice^1^, rabbits^2^, cows^3^, and humans^4^, and has enabled several successful human pregnancies and childbirths^5^,^6^. Compared with traditional cryopreservation methods, vitrification is more effective, easier to perform, and less time-consuming. However, vitrification can have adverse effects on oocytes such as spindle confusion, zona pellucida (ZP) hardening, and death, mainly due to the use of high concentrations of cryoprotectants that have a toxic effect on cells. The magnitude of this toxic effect is related to the duration of cell exposure to cryoprotectant, the temperature at which cells are equilibrated in cryoprotectant, the speed at which temperature decreases to −196 °C, and the size of cells. The relatively large size of mammalian oocytes (~80 μm for mouse oocytes) makes the genetic material inside cells more susceptible to damage by temperature changes and chemical reagents. Specifically, spindle damage occurs after oocytes rapidly freeze and thaw^7^. Moreover, vitrification alters microfilament structure, which can be repaired 3 hours after thawing^8^. These observations suggest that vitrification outcomes could be improved by modifying cooling and warming conditions. In this study, we tested the effects of different vitrification parameters related to cryoprotectant composition, temperature and duration of exposure to vitrification, thawing, and fertilization solutions, presence of cumulus layers, and carrier material on oocyte survival, fertilization, and blastocyst formation. Determining the optimal specifications for each step could improve the use of vitrification for oocyte cryopreservation.

## Results

### Toxic effect of different cryoprotectants and concentration on oocyte survival, fertilization, and blastocyst formation

**Figure 1:**
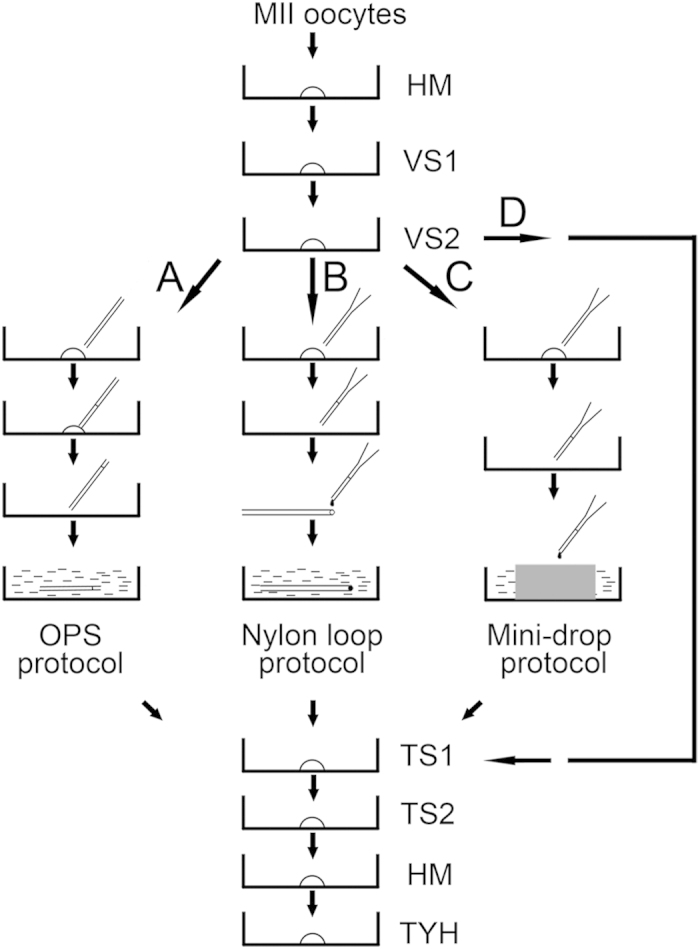
Oocyte vitrification process. Mature mouse oocytes were sequentially transferred to holding medium (HM), vitrification solution 1 (VS1), and vitrification solution 2 (VS2). After loading with different carriers, oocytes were immersed in liquid nitrogen. Oocytes were thawed by sequential transfer to thawing solution 1 (TS1), thawing solution 2 (TS2), and HM. After incubation in fertilization medium (Toyoda-Yokoyama-Hosoki medium (TYH)), oocytes were subjected to insemination. (**A**) Open pulled straw (OPS) protocol. (**B**) Nylon loop protocol. (**C**) Mini-drop protocol. (**D**) Procedure without freezing.

Procedures for oocyte cryopreservation were summarized in Fig. 1. For oocyte equalization in VS prior to freezing, glycerin (GLY), ethylene glycol (EG), dimethylsulfoxide (Me2SO), or propylene glycol (PrOH) was added to the VS to serve as cryoprotectant. Six types of cryoprotectants added to VS1 and VS2 were tested: 20% (v/v) GLY (VS1) and 40% GLY (VS2), 20% EG (VS1) and 40% EG (VS2), 20% Me2SO (VS1) and 40% Me2SO (VS2), 20% PrOH (VS1) and 40% PrOH (VS2), 10% GLY + 10% PrOH (VS1) and 20% GLY + 20% PrOH (VS2), and 10% EG + 10% Me2SO (VS1) and 20% EG + 20% Me2SO (VS2).

Mature OCCs were equilibrated in VS1 for 2 mins and VS2 for 20 secs prior to incubation in TS1 (M199 + 20% FCS + 0.33 mol/l sucrose) for 3 mins and TS2 (M199 + 20% FCS + 0.25 mol/l sucrose) for 3 mins. For the control group, OCCs were incubated in HM followed by incubation in TS1 and TS2. All procedures were conducted at room temperature. After three washes in TYH medium, all oocytes were subjected to insemination and embryo culture.

**Figure 2:**
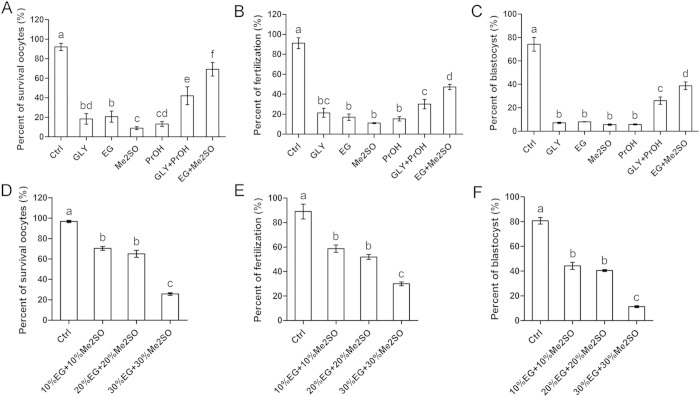
Toxic effect of different cryoprotectants and concentration on OCCs. GLY, EG, Me2SO, PrOH, GLY + PrOH, and EG + Me2SO cryoprotectants were tested. A group without cryoprotectant was used as a control. (**A**) Oocyte survival. (**B**) Fertilization. (**C**) Blastocyst formation. Cryoprotectants containing 10% EG + 10% Me2SO, 20% EG + 20% Me2SO, or 30% EG + 30% Me2SO were tested. A group without cryoprotectant was used as a control. (**D**) Oocyte survival. (**E**) Fertilization. (**F**) Blastocyst formation. Different superscript letters (a–f) indicate *p* < 0.01.

We found that oocytes exposed to EG + Me2SO exhibited greater survival (69.2 ± 7.0%; Fig. 2A), fertilization (47.3 ± 2.7%; Fig. 2B), and blastocyst formation (38.8 ± 3.2%; Fig. 2C) than those exposed to GLY (18.4 ± 5.4% survival, 21.4 ± 4.4% fertilization, and 7.1 ± 0.6% blastocyst formation), EG (20.7 ± 5.8% survival, 17.0 ± 3.2% fertilization, and 8.0 ± 0.2% blastocyst formation), Me2SO (8.9 ± 1.5% survival, 11.1 ± 0.5% fertilization, and 5.6 ± 0.6% blastocyst formation), PrOH (13.1 ± 2.5% survival, 15.4 ± 2.2% fertilization, and 5.8 ± 0.5% blastocyst formation), or GLY + PrOH (42.1 ± 9.1% survival, 30.1 ± 4.7% fertilization, and 26.1 ± 3.1% blastocyst formation). Oocytes in the control group showed greater survival (92.1 ± 3.6%), fertilization (91.2 ± 5.5%), and blastocyst formation (74.2 ± 5.9%) than all other groups. Based on these results, EG + Me2SO was used as the cryoprotectant in the following experiments.

Next, we evaluated the toxic effect of different concentrations of EG + Me2SO in VS2. VS1 was supplemented with 10% EG + 10% Me2SO, and VS2 was supplemented with 10% EG + 10% Me2SO, 20% EG + 20% Me2SO, or 30% EG + 30% Me2SO. The other steps were the same as those previously described. We found that oocytes equilibrated in VS2 containing 10% EG + 10% Me2SO or 20% EG + 20% Me2SO showed similar survival (70.3 ± 1.8% and 65.0 ± 3.5%, respectively; Fig. 2D), fertilization (58.7 ± 3.0% and 51.9 ± 2.0%, respectively; Fig. 2E), and blastocyst formation (44.3 ± 2.8% and 40.5 ± 0.9%, respectively; Fig. 2F). Compared with these two groups, oocytes equilibrated in VS2 containing 30% EG + 30% Me2SO exhibited lower survival (25.7 ± 1.2%), fertilization (30.0 ± 1.6%), and blastocyst formation (11.3 ± 0.9%), and oocytes in the control group exhibited higher survival (96.8 ± 1.2%), fertilization (89.0 ± 6.1%), and blastocyst formation (80.7 ± 2.7%). Therefore, VS2 with a concentration of 20% EG + 20% Me2SO was used in the following experiments.

### Effect of concentration of FCS on oocyte survival, fertilization, and blastocyst formation

**Figure 3:**
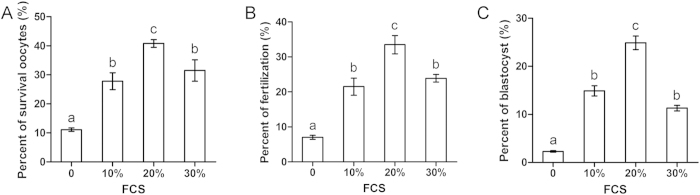
Effect of concentrations of fetal calf serum (FCS) on OCCs. A concentrations of 0%, 10%, 20%, or 30% FCS was supplemented into VS. (**A**) Oocyte survival. (**B**) Fertilization. (**C**) Blastocyst formation. Different superscript letters (a–c) indicate *p* < 0.01.

We next investigated whether different concentrations of FCS in VS affect oocyte cryopreservation. TCM199 supplemented with 0%, 10%, 20%, or 30% FCS was used as basic solution for preparing VS1 (10% EG + 10% Me2SO) and VS2 (20% EG + 20% Me2SO). At room temperature, OCCs were equilibrated in VS1 for 2 mins and VS2 for 20 secs. Samples were loaded into OPSs immediately after equilibration in VS2 and placed into liquid nitrogen. After storing in liquid nitrogen for 1 week, OCCs were recovered by incubation in TS1 and TS2. All other steps were the same as those previously described. We found that the 20% FCS group displayed the greatest survival (40.8 ± 1.3%; Fig. 3A), fertilization (33.5 ± 2.6%; Fig. 3B), and blastocyst formation (24.9 ± 1.4%; Fig. 3C). The 10% and 30% FCS groups exhibited lower survival (27.8 ± 2.9% and 31.5 ± 3.7%, respectively), fertilization (21.5 ± 2.4% and 23.9 ± 1.1%, respectively), and blastocyst formation (14.9 ± 1.1% and 11.3 ± 0.6%, respectively). The control group without FCS exhibited the lowest survival (11.1 ± 0.6%), fertilization (7.0 ± 0.6%), and blastocyst formation (2.3 ± 0.2%). Thus, a concentration of 20% FCS in VS was used in the following experiments.

### Effect of duration of equilibration in VS1, VS2 and TS on oocyte survival, fertilization, and blastocyst formation

**Figure 4:**
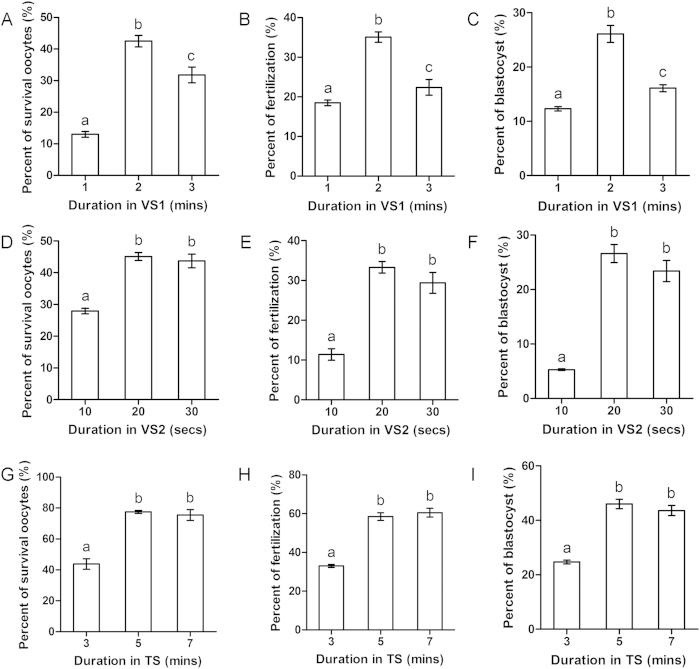
Effect of duration of equilibration in VS1, VS2 and thawing solution (TS) on OCCs. Oocytes were equilibrated in VS1 for 1 min, 2 min, or 3 min followed by equilibration in VS2. (**A**) Oocyte survival. (**B**) Fertilization. (**C**) Blastocyst formation. Oocytes were equilibrated in VS1 and then equilibrated in VS2 for 10 secs, 20 secs, or 30 secs. (**D**) Oocyte survival. (**E**) Fertilization. (**F**) Blastocyst formation. After freezing, oocytes were incubated in TS for 3 min, 5 min, or 7 min. (**G**) Oocyte survival. (**H**) Fertilization. (**I**) Blastocyst formation. Different superscript letters (a–c) indicate *p* < 0.01.

In this experiment, we examined the effect of the duration of equilibration in VS1 on oocyte vitrification. VS1 containing M199 + 20% FCS + 10% EG + 10% Me2SO and VS2 containing M199 + 20% FCS + 20% EG + 20% Me2SO were used as cryoprotectants. OCCs were equilibrated in VS1 for 1 min, 2 mins, or 3 mins. All other procedures were the same as those previously described. We found that the 2-min group displayed the greatest survival (42.5 ± 1.8%; Fig. 4A), fertilization (35.1 ± 1.3%; Fig. 4B), and blastocyst formation (26.1 ± 1.6%; Fig. 4C). The 3-min group showed lower survival (31.8 ± 2.5%), fertilization (22.4 ± 2.0%), and blastocyst formation (16.1 ± 0.6%), and the 1-min group showed the lowest survival (13.0 ± 0.9%), fertilization (18.5 ± 0.7%), and blastocyst formation (12.3 ± 0.4%). Thus, an equilibration duration of 2 mins in VS1 was used in the following experiments.

We next tested the effect of the duration of equilibration in VS2 on oocyte vitrification. OCCs were equilibrated in VS1 for 2 mins and then equilibrated in VS2 for 10 secs, 20 secs, or 30 secs. All other procedures were the same as those previously described. The 20-sec and 30-sec groups showed equivalently high survival (45.1 ± 1.3% and 43.7 ± 2.2%, respectively; Fig. 4D), fertilization (33.3 ± 1.5% and 29.4 ± 2.6%, respectively; Fig. 4E), and blastocyst formation (26.6 ± 1.7% and 23.4 ± 1.9%, respectively; Fig. 4F), but the 10-sec group showed lower survival (27.9 ± 0.9%), fertilization (11.4 ± 1.5%), and blastocyst formation (5.3 ± 0.2%). We therefore used an equilibration duration of 20 secs in VS2 in the following experiments.

Next, we assessed how the duration of incubation in TS1 and TS2 affects OCC vitrification. OCCs were equilibrated for 2 mins in VS1 and 20 secs in VS2. After recovery from liquid nitrogen, OCCs were incubated in TS1 and TS2 for 3 mins, 5 mins, or 7 mins. All other steps were the same as those previously described. The 5-min and 7-min groups showed similarly high survival (77.5 ± 0.9% and 75.5 ± 3.4%, respectively; Fig. 4G), fertilization (58.5 ± 2.0% and 60.5 ± 2.3%, respectively; Fig. 4H), and blastocyst formation (46.0 ± 1.7% and 43.6 ± 1.9%, respectively; Fig. 4I), whereas the 3-min group showed lower survival (43.8 ± 3.4%), fertilization (33.0 ± 0.8%), and blastocyst formation (24.7 ± 0.7%). Thus, an incubation time of 5 mins in TS1 and TS2 was used in the following experiments.

### Effect of temperature of exposure to VS and TS on oocyte survival, fertilization, and blastocyst formation

**Figure 5:**
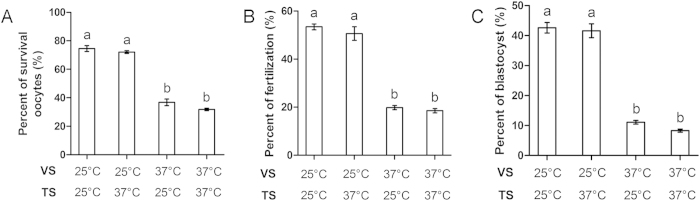
Effect of temperature of exposure to VS and TS on OCCs. Oocytes were exposed to different temperatures of VS (25 °C or 37 °C) and TS (25 °C or 37 °C). (**A**) Oocyte survival. (**B**) Fertilization. (**C**) Blastocyst formation. Different superscript letters (a,b) indicate *p* < 0.01.

Next, we tested the effect of temperature of exposure to VS and TS on OCC vitrification. OCCs were exposed to VS at 25 °C and TS at 25 °C, VS at 25 °C and TS at 37 °C, VS at 37 °C and TS at 25 °C, or VS at 37 °C and TS at 37 °C. All other procedures were same as those previously described. We found no differences between the two groups exposed to VS at 25 °C (survival: 74.6 ± 2.0% and 72.1 ± 1.0%; fertilization: 53.4 ± 1.2% and 50.6 ± 2.8%; blastocyst formation: 42.6 ± 1.8% and 41.6 ± 2.3%, respectively; Fig. 5A–C). Similarly, there were no differences between the two groups exposed to VS at 37 °C (survival: 36.8 ± 2.3% and 31.8 ± 0.9%; fertilization: 19.8 ± 0.9% and 18.5 ± 0.9%; blastocyst formation: 11.1 ± 0.6% and 8.3 ± 0.5%, respectively). However, groups exposed to VS at 25 °C showed greater oocyte survival, fertilization, and blastocyst formation than groups exposed to VS at 37 °C. Thus, OCCs were exposed to VS and TS at 25 °C in the following experiments.

### Effect of cumulus layer presence on oocyte survival, fertilization, and blastocyst formation

**Figure 6:**
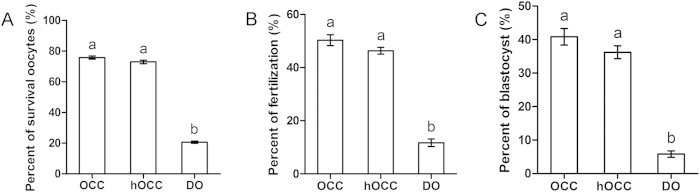
Effect of cumulus layer presence on OCCs. OCCs were treated with hyaluronidase to disperse cumulus layers to obtain half OCCs (hOCCs) or denuded oocytes (DOs). (**A**) Oocyte survival. (**B**) Fertilization. (**C**) Blastocyst formation. Different superscript letters (a,b) indicate *p* < 0.01.

We next evaluated the contribution of the number of cumulus layers on oocyte vitrification. Equilibration in VS and incubation in TS was conducted at 25 °C. Oocytes with at least four cumulus layers (denoted as OCC), approximately half of the cumulus layers intact (denoted as hOCC), or without cumulus layers (denoted as denuded oocytes (DO)) were subjected to vitrification. All other procedures were the same as those previously described. We found no differences between OCC and hOCC groups in survival (75.8 ± 1.0% and 73.0 ± 1.1%, respectively; Fig. 6A), fertilization (50.3 ± 2.1% and 46.3 ± 1.3%, respectively; Fig. 6B), or blastocyst formation (40.8 ± 2.5% and 36.2 ± 1.9%, respectively; Fig. 6C). However, DOs showed lower survival (20.6 ± 0.8%), fertilization (11.7 ± 1.4%), and blastocyst formation (5.8 ± 1.0%). Thus, fully intact OCCs were used in the following experiments.

### Effect of duration of incubation in TYH medium on oocyte survival, fertilization, and blastocyst formation

**Figure 7:**
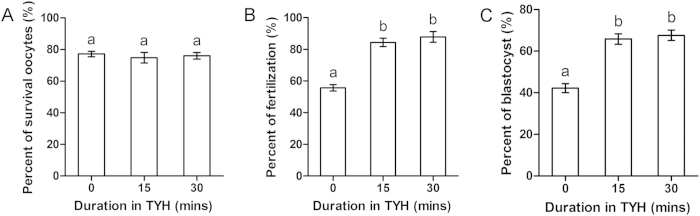
Effect of incubation in TYH medium on OCCs. After thawing, OCCs were incubated in TYH medium for 0 min, 15 min, or 30 min. (**A**) Oocyte survival. (**B**) Fertilization. (**C**) Blastocyst formation. Different superscript letters (a,b) indicate *p* < 0.01.

In this experiment, we assessed the effect of incubation duration in TYH medium immediately after intact OCCs were transferred from TS. OCCs were incubated in TYH for 0 min, 15 mins, or 30 mins. Insemination was conducted after incubation of OCCs in TYH. All other procedures were the same as those previously described. We found no differences in survival between the three groups (77.2 ± 1.7%, 74.8 ± 3.3%, and 76.0 ± 2.1%; Fig. 7A). However, fertilization (Fig. 7B) and blastocyst formation (Fig. 7C) were higher in the 15-min and 30-min groups than in the 0-min group (fertilization: 84.4 ± 2.6%, 87.9 ± 3.4%, and 55.7 ± 2.1%, respectively; blastocyst formation: 65.8 ± 2.5%, 67.6 ± 2.5%, and 42.2 ± 2.1%, respectively). Thus, OCCs were incubated in TYH for 30 mins in the following experiments.

### Effect of different oocyte carriers on oocyte survival, fertilization, and blastocyst formation

**Figure 8:**
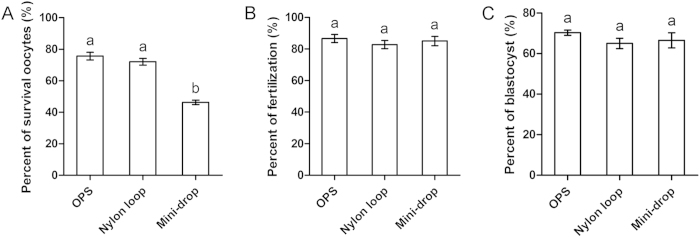
Effect of different carriers on OCCs. Open pulled straw (OPS), nylon loop, and mini-drop were used to load oocytes for cryopreservation. (**A**) Oocyte survival. (**B**) Fertilization. (**C**) Blastocyst formation. Different superscript letters (a,b) indicate *p* < 0.01.

Next, we tested three types of oocytes carriers: OPS (Fig. 1A), nylon loop (Fig. 1B), and mini-drop (Fig. 1C). After transferring from TS, oocytes were incubated in TYH for 30 mins prior to insemination. All other procedures were the same as those previously described. We found no differences among these three methods for fertilization (86.6 ± 2.6%, 82.8 ± 2.6%, and 85.0 ± 3.0%, respectively; Fig. 8A) or blastocyst formation (70.3 ± 1.3%, 65.0 ± 2.6%, and 66.5 ± 3.7%, respectively; Fig. 8B). However, OPS and nylon loop methods were associated with greater survival than the mini-drop method (75.6 ± 2.4%, 72.1 ± 2.1%, and 46.3 ± 1.4%, respectively; Fig. 8C). Therefore, we conclude that OPS and nylon loop methods are more suitable than the mini-drop method for oocyte vitrification.

### Combinations of optimized key parameters improved oocyte spindle morphology

**Figure 9:**
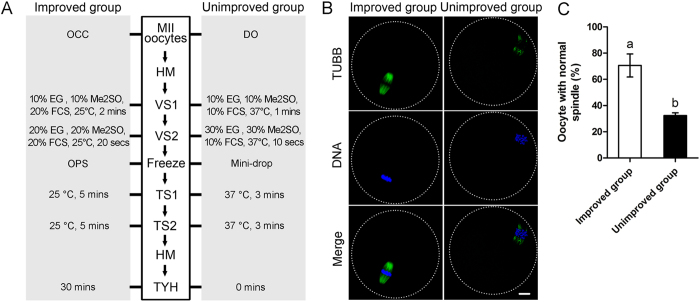
Spindles morphology was improved by employing the optimized key parameters during vitrification. (**A**) Different vitrification procedure used in improved and unimproved groups. (**B**) Representative images of spindles in improved and unimproved group. Green: TUBB; Blue: DNA; Merge: overlay of TUBB and DNA. Scale bar = 10 μm. (**C**) Oocytes in improved group get higher percent of normal spindles than that in unimproved group. Different superscript letters (a,b) indicate *p* < 0.01.

Procedures of freezing and thawing can damage oocyte spindle, which may affect oocyte survival, fertilization, and embryo development. In our study, spindles of oocytes from improved and unimproved vitrification combinations were compared. In the improved group, the best conditions at each steps were used for oocytes cryopreservation; whereas in the unimproved group, the worst conditions at each steps were used for oocytes cryopreservation (Fig. 9A). After thawing followed by 3 hours incubation in CZB medium, most of the spindles reappeared (Fig. 9B). Our results showed that improved group have higher percent of normal spindle than that in the unimproved group (70.6 ± 8.8% and 32.4 ± 2.1%, *p* < 0.05, Fig. 9C).

## Discussion

Cryoprotectant is the most important component of successful vitrification. High concentrations of cryoprotectant inside cells become hyaloid material under ultra-cooling conditions, which prevents the formation of ice crystals that are harmful to cells. However, a high concentration of cryoprotectant has toxic effects that may lead to oocyte damage. Thus, both permeability and toxicity need to be considered when determining the optimal concentration of cryoprotectant. Many types of cryoprotectants, such as Me2SO, PrOH, and EG, have been used for oocyte cryopreservation^9^,^10^,^11^. A previous study reports that EG has the lowest toxicity^12^, whereas Me2SO causes ZP hardening and prevents fertilization^13^,^14^. Some studies suggest that using a mixed reagent containing different cryoprotectants exerts less toxicity^15^. In the present study, we observed high rates of survival, fertilization, and embryo development when cryoprotectant consisted of EG and Me2SO. Furthermore, this combination of cryoprotectant was less toxic to oocytes than each cryoprotectant used separately. Although GLY and PrOH are often used as cryoprotectants, their permeabilities are not as good as those of EG and Me2SO. Because a high concentration of cryoprotectant does not allow long periods of equilibration in VS, the permeation of GLY and PrOH into cells is insufficient. Therefore, a combination of GLY and PrOH is not suitable for oocyte vitrification.

Although a higher concentration of cryoprotectant results in better dehydration of cells, the ability of oocytes to endure toxicity should also be considered. In other words, finding a balance between high cryoprotectant concentration and low toxicity is key to successful vitrification. We found that a total of concentration of 40% was somewhat toxic to oocytes, but a total concentration of 20% did not lessen the toxic effects on oocyte survival and embryo development. Thus, a concentration of 40% appears to be optimal for successful vitrification. A recent study in pigs shows that cryoprotectant composed of 15% Me2SO and 15% EG improved the *in vivo* development of vitrified mature oocytes^16^. However, the amount of lipids and the size of oocytes differ substantially between mice and pigs, which may explain differences between species in the optimal concentration of cryoprotectant.

After vitrification, oocytes exhibit poor capacity for fertilization due to ZP hardening. Previous studies report that oocytes vitrified in VS containing FCS show a comparable fertilization rate as control oocytes without VS treatment^17^,^18^,^19^. This is because the existence of FCS in the perivitelline space extrudes residual cryoprotectant, resulting in increased fertilization. Also, FCS has been found to prevent ZP hardening through its fetuin content^20^. We found that the removal of FCS from VS worsened the outcomes of oocyte vitrification. Also, our results suggest that using 10% FCS is probably not sufficient to prevent ZP hardening. Surprisingly, 20% FCS was associated with the highest rate of oocyte survival, fertilization, and blastocyst formation, and 30% FCS did not produce better results. Therefore, higher concentrations of FCS do not appear to benefit the vitrification process. Another reason responsible for this is that high FCS can prevent cryoprotectant from permeating into oocytes, and then leads to the formation of ice crystals and damage oocytes.

The reduction of equilibration time in VS is an effective way to reduce the damage caused by cryoprotectant^21^. However, larger oocytes require longer exposure to VS for full dehydration. To resolve this conflict, we employed a two-step equilibration protocol, which allows sufficient time for oocytes to dehydrate but also minimizes the harm of high VS concentration. We found that an equilibration time of 2 mins in VS1 and 20 or 30 secs in VS2 resulted in the greatest survival, fertilization, and blastocyst formation, whereas less time in VS (i.e., 1 min in VS and 10 secs in VS2) may not be sufficient to permeate oocytes. However, our results also suggest that longer exposure to cryoprotectant may do more harm than good.

The process of oocyte thawing involves an increase in temperature, exclusion of cryoprotectant inside cells, and rehydration. Incorrect thawing methods can lead to reformation of ice crystals and damage to the subcellular structure of oocytes. In this context, osmotic pressure is a key factor affecting vitrification outcomes. Including sucrose in the TS provides a higher osmotic pressure that minimizes the rapid movement of water^21^. Also, a sufficient length of time is necessary for substituting TS for VS. We obtained the best results by employing two steps of VS removal and found that an incubation time of less than 3 mins in TS was not sufficient to completely remove VS, which had a negative effect on oocyte survival and embryo development.

The temperature at which oocytes are exposed to VS and TS also affects vitrification outcomes. Higher temperatures are associated with greater toxic effects of cryoprotectant due to sudden changes in osmotic pressure. Lower temperature can reduce the toxicity of cryoprotectants^22^. We found that a VS temperature of 25 °C allowed cryoprotectant to infiltrate oocytes quickly with less toxicity. However, when the temperature was increased to 37 °C, oocytes could not endure the faster change in osmotic pressure, resulting in lower survival and compromised developmental potential. As for the temperature of TS, both 25 °C and 37 °C resulted in good rates of survival and embryo development. However, a recent study reported that human oocytes rehydrated at 37 °C get higher survival than those rehydrated at room temperature^23^. Inconformity of results may due to the differences of cryoprotectants, freeing and warming time, especially species. Moreover, except choosing a suitable temperature for freezing and warming, the cooling and warming rates maybe important. Recently, a study on human oocyte reported that they have determined freezing and warming rates using a data logger. A novel, aseptic closed system vitrification technique for the cryopreservation of embryos and oocytes has been developed and clinically validated in there study^24^.

Although a previous study reports that cumulus cells do not contribute to oocyte survival after freezing^25^, accumulating data suggest that cumulus cells and their extracellular matrix promote fertilization^26^,^27^. We found that oocytes surrounded by fully intact or partial cumulus layers had a higher fertilization rate than denuded oocytes, which is consistent with previous results^28^. Based on these findings, we conclude that the presence of cumulus layers promotes oocyte survival after freezing. We hypothesize that VS-induced changes in osmotic pressure inside oocytes can be minimized by the presence of cumulus cells. The smaller size of cumulus cells compared with oocytes may enable cumulus cells to tolerate high concentrations of cryoprotectant, although this possibility needs further investigation.

Cytoskeletal structure is very sensitive to temperature changes. A study by Eroglu *et al.* showed that incubation of oocytes in fertilization medium for 1 hour after freezing rescues cytoskeletal disruption, which benefits fertilization and embryo development^29^. In our study, oocytes incubated in TYH for 15 or 30 mins showed increased fertilization and embryo development, although this step did not promote oocyte survival. We propose that recovery in fertilization medium for a short time before fertilization allows the reorganization of tubulin and microfilament components inside oocytes, which in turn leads to better fertilization and embryo development. Similar to our results, some studies in human reported that spindles recovered for a period of time after vitrification improve the embryo development^30^,^31^ and this recovery may make the vitrified oocyte has comparable cleavage timings, cell number, and DNA methylation patterns with the freshed oocytes^32^.

A key step of vitrification is the rapid decrease in temperature of tissue. In the present study, we compared three types of previously described carriers for oocyte vitrification^16^,^33^,^34^. We found no differences among carriers in terms of fertilization and embryo development. However, OPS and nylon loop methods resulted in greater survival than the mini-drop method. OPS and nylon loop methods were performed by quickly placing oocytes into liquid nitrogen, whereas the mini-drop method was performed by dropping liquid onto the surface of iron at a temperature of −196 °C. Thus, the greater survival of oocytes frozen with OPS and nylon loop methods may be due to the faster decrease in temperature. In addition, using the mini-drop method, it was difficult to recover oocytes from the storage tank, making this method not very applicable for oocyte cryopreservation.

In summary, our results demonstrate that the composition and concentration of cryoprotectant, duration and temperature of exposure to VS, TS, and TYH, presence of cumulus cells, and type of carrier affects the outcomes of mouse oocyte vitrification. The best outcomes can be obtained by using an optimized vitrification protocol.

## Materials and Methods

### Ethics statement

All studies adhered to procedures consistent with the National Research Council Guide for the Care and Use of Laboratory Animals and were approved by the Institutional Animal Care and Use Committee at Inner Mongolia University.

### Oocyte collection

Oocytes were collected from adult (8–12 weeks of age) F1 female B6D2 mice. All chemicals and media were purchased from Sigma-Aldrich (St. Louis, MO, USA) unless stated otherwise. For *in vivo* metaphase II (MII) stage oocyte-cumulus complex (OCC) collection, superovulation was induced by 7 IU pregnant mare serum gonadotropin (SanSheng, Ningbo, China) followed by 7 IU human chorionic gonadotropin (SanSheng) 48 hours later. In some experiments, cumulus cells were dispersed by 0.3 mg/ml hyaluronidase in HEPES-M2 medium.

### Oocyte freezing and thawing

Oocytes subjected to cryopreservation were transferred to holding medium (HM; TCM199 + 20% (v/v) fetal calf serum (FCS)) prior to sequential equilibration in vitrification solution 1 (VS1) and VS2. The gentle mixture of oocytes with VS occurred immediately after transfer to ensure adequate equilibrium. After equilibration in VS2, oocytes were loaded into one of three types of carriers: open pulled straw (OPS; Fig. 1, path A), nylon loop (Fig. 1; path B), or mini-drop (Fig. 1; path C). OPSs were made in-house by polishing commercial plastic tubes (Tianshankaifeng Company, Beijing, China) into thin-walled tubes (outer diameter = 200 μm, inner diameter = 180 μm) with fire. Nylon loops were made by fixing fine nylon onto iron wires. Mini-drop was carried out by dropping liquid at a volume of less than 20 μl onto an iron surface immersed in liquid nitrogen.

For experiments without freezing, oocytes were transferred from VS2 directly to thawing solution 1 (TS1; Fig. 1, path D). Oocytes subjected to freezing were rapidly placed into liquid nitrogen with their carriers. After 1 week of storage in liquid nitrogen, carriers were collected and immediately transferred to TS1. After incubation with TS1, oocytes were observed under an inverted microscope, transferred to TS2, and then placed in HM. Temperature control during exposure to VS or TS was conducted using MSP Selectable Temperature (Thermo Plate, Japan). Thawed oocytes were then transferred from HM to Toyoda Yokoyama Hosoki (TYH) medium (Fig. 1).

### *In vitro* fertilization and embryo culture

Adult (12–14 weeks of age) male B6D2 F1 mice were used for sperm collection. Sperm suspension collected from the epididymis was held for 2 hours in 200 μl TYH medium supplemented with 4 mg/ml bovine serum albumin (BSA). Recovered MII oocytes were incubated with spermatozoa for 6 hours in 200 μl TYH medium supplemented with 4 mg/ml BSA. The final sperm concentration for fertilization was 1 × 10^6^/ml. Zygotes were cultured in Chatot-Ziomet-Bavister (CZB) medium without glucose in a humidified atmosphere of 5% CO_2_ at 37 °C for the first 2 days and then transferred to CZB medium supplemented with 5.5 mmol/l glucose when embryos reached the four-cell stage. Embryo development was observed 24 and 96 hours after fertilization to calculate the percentage of fertilization and blastocyst formation, respectively.

### Immunofluorescence and Confocal Microscopy

After thawing, oocytes were incubated in CZB medium for 3 hours to allow reformation of spindle. Oocytes were then fixed with 4% paraformaldehyde for 40 mins and then permeabilized with 0.5% Triton X-100 for 2.5 hours. Following blocking in 1% BSA in PBS containing 1/1,000 Tween-20 and 1/10,000 Triton X-100 for 1 hour. Then samples were incubated overnight at 4 °C with mouse anti-TUBB antibody (Abcam). Chromosomes were stained with DAPI (5μg/ml, Roche, Mannheim, Germany) for 10 mins. After staining, samples were mounted on glass slides using vectashield (Vector Labs, Burlingame, CA) mounting medium and examined with a confocal laser scanning microscope (Nikon, A1R, Japan).

### Statistical analysis

The total number of oocytes for each group was at least 400. Data are presented as mean + SEM (n = 3). Differences among groups in survival (number of surviving oocytes/number of recovered oocytes), fertilization (number of two-cell zygotes/number of surviving oocytes), and blastocyst formation (number of blastocysts/number of surviving oocytes) was evaluated using χ^2^ tests. A *p*-value < 0.05 was considered statistically significant.

## Additional Information

**How to cite this article**: Zhou, C.-J. *et al.* High survival of mouse oocytes using an optimized vitrification protocol. *Sci. Rep.* **6**, 19465; doi: 10.1038/srep19465 (2016).
